# Environmental Factor Index (EFI): A Novel Approach to Measure the Strength of Environmental Influence on DNA Methylation in Identical Twins

**DOI:** 10.3390/epigenomes8040044

**Published:** 2024-11-21

**Authors:** Yoichi Takenaka, Osaka Twin Research Group, Mikio Watanabe

**Affiliations:** 1 Faculty of Informatics, Kansai University, Osaka 569-1052, Japan; 2 Center for Twin Research, Graduate School of Medicine, The University of Osaka, Osaka 565-0871, Japannabe@sahs.med.osaka-u.ac.jp (M.W.); 3 Department of Clinical Laboratory and Biomedical Sciences, Graduate School of Medicine, The University of Osaka, Osaka 565-0871, Japan

**Keywords:** epigenetics, DNA methylation, environmental factors, twin study

## Abstract

Background/Objectives: The dynamic interaction between genomic DNA, epigenetic modifications, and phenotypic traits was examined in identical twins. Environmental perturbations can induce epigenetic changes in DNA methylation, influencing gene expression and phenotypes. Although DNA methylation mediates gene-environment correlations, the quantitative effects of external factors on DNA methylation remain underexplored. This study aimed to quantify these effects using a novel approach. Methods: A cohort study was conducted on healthy monozygotic twins to evaluate the influence of environmental stimuli on DNA methylation. We developed the Environmental Factor Index (EFI) to identify methylation sites showing statistically significant changes in response to environmental stimuli. We analyzed the identified sites for associations with disorders, DNA methylation markers, and CpG islands. Results: The EFI identified methylation sites that exhibited significant associations with genes linked to various disorders, particularly cancer. These sites were overrepresented on CpG islands compared to other genomic features, highlighting their regulatory importance. Conclusions: The EFI is a valuable tool for understanding the molecular mechanisms underlying disease pathogenesis. It provides insights into the development of preventive and therapeutic strategies and offers a new perspective on the role of environmental factors in epigenetic regulation.

## 1. Introduction

DNA methylation is an important epigenetic component. Although all of the cells in multicellular organisms have identical genomes, differentiated cells, such as those in the liver and muscle, appear different, with varied roles; however, the phenotypes at the cellular level may differ within the same genome (DNA sequence). This concept is known as epigenetics and is defined as a “stably heritable phenotype resulting from changes in a chromosome without alterations in the DNA sequence” [1].

DNA methylation is the process in which methyl groups are added primarily to cytosine residues of genomic DNA [2,3,4,5,6]. The methylation state of the genome is inherited from cell division [7,8] and plays an important role in normal development and cell differentiation in vertebrates [9,10]. Interestingly, DNA methylation alters gene expression, and methylation occurring in the promoter regions inhibits DNA binding to transcription factors [11], thus decreasing their expression. Conversely, the methylation of gene bodies promotes transcription [12,13]. These phenomena maintain the tissue- or cell-type-specific function of differentiated cells. Therefore, it can be inferred that DNA methylation stably alters gene expression profiles to remember its location/position in the cell differentiation tree [14].

DNA methylation was recently identified as the core of the etiology of human disorders. The dysregulation of genes resulting from DNA methylation causes various neurodevelopmental syndromes, such as fragile X and Rett, immunodeficiency, centromeric region instability, and facial anomalies (ICF) [15]. Abnormal DNA methylation is also associated with breast [16,17], cervical [16,18], and other cancers [19,20,21,22,23,24], as well as diabetes [25,26,27], kidney disease [28,29], autoimmune disorders [30,31,32], and allergies [33,34]. Moreover, the rate of cell aging can be quantified based on DNA methylation levels [35,36,37,38,39].

Because the correlation between a single DNA methylation site and the resulting phenotype is low, many DNA methylation sites are associated with various phenotypes. For example, the number of sites needed to measure cell age was 353 for the Horvath clock [35,39], 71 for the Hannum epigenetic age [36], and 513 for the Levine clock [38]. These numerous methylation sites resulted from an abundance of positions with methylation potential. The human genome contains >20 million CpG dinucleotide sequences that are methylation candidates. Therefore, machine learning has proven to be a valuable tool to determine these associations.

Genetic and environmental factors can have an impact on DNA methylation, which in turn, affects the phenotype. Genetic factors are determined solely by inheritance, whereas environmental factors include climate, nutrition, lifestyle, stress, drugs, and living conditions. The combined influence of genetic and environmental factors can be observed in various phenotypic outcomes. For example, monogenic diseases arise from mutations in a single gene, whereas dietary habits are considered one of the causes of colorectal cancer [40]. This effect on the initiation and progression of various diseases has also been demonstrated through insurance claims [41] and studies on twins [42].

DNA methylation is an important mechanism that connects genetic and environmental factors to a particular phenotype. For example, exposure to chemicals, such as polychlorinated biphenyls, lead, and bisphenol A, has been associated with an increased risk of autism spectrum disorders, potentially through alterations in DNA methylation [43]. In addition, children can inherit parental stress [44] and nutritional status [45] through DNA methylation. Epigenomic changes induced by environmental factors also influence the development of more diseases compared with genetic elements [46]. Thus, determining the effects of genetic and environmental factors on DNA methylation will improve our understanding of the etiology and pathogenesis of these diseases [47].

Should the contributions of environmental and genetic factors to DNA methylation be successfully separated, this could significantly aid in unraveling the causes of diseases and developing novel therapeutic approaches. Fujii found that DNA methylation is a mediator of associations between environment and diseases [48]. However, the interplay between genetic and environmental factors in DNA methylation denotes a complex mechanism, making separating their respective contributions challenging from biological and technical perspectives [49,50] except when using monozygotic twins.

Monozygotic twins share the same genetic material and are used to differentiate the effects of genetic and environmental factors on DNA methylation levels [51,52]. Based on this unique feature, twin studies enhance our understanding of the processes regulating epigenetic variation and unravel the relative contributions of environmental and genetic factors to complex traits [53,54]. Building upon these findings, we proposed an index to measure the influence of environmental factors on each methylation site. This index compares the differences in methylation levels between younger twins and elderly twins. Overall, our analysis provides insight into the complex interplay between DNA methylation and environmental factors that have implications for disease development and prevention.

To address the gap in the understanding of the influence of environmental stimuli on DNA methylation, we introduced the Environmental Factor Index (EFI) in this study. We designed the EFI to identify the methylation sites that show statistically significant changes in response to environmental stimuli. By comparing the methylation patterns between younger and elderly twin pairs, we aim to quantify the influence of environmental factors on specific methylation sites. This approach provides insights into the potential regulatory mechanisms and the association of methylation changes with various disorders, particularly those linked to CpG islands. The EFI represents a novel tool for advancing our understanding of the molecular mechanisms underlying disease pathogenesis and developing preventive and therapeutic strategies.

Current methods, such as a correlation analysis between methylation levels and age and epigenome-wide association studies (EWAS) to assess environmental impacts for analyzing the impact of environmental factors on DNA methylation often fail to effectively differentiate the influence of genetic factors from environmental stimuli. Traditional approaches may lack the statistical power or specificity to identify subtle yet significant epigenetic changes linked to environmental factors. We explicitly designed the EFI to address these challenges by utilizing monozygotic twins, thereby more precisely isolating the impact of environmental factors.

Unlike previous metrics, the EFI provides a novel approach that focuses on isolating the specific environmental contributions to methylation changes, which is particularly crucial in understanding disease mechanisms. By comparing younger and older twin pairs, the EFI offers a unique lens through which we can observe the cumulative impact of environmental exposure over time, something that previous studies have struggled to accurately quantify.

## 2. Results

### 2.1. Difference Between EFI and Correlation Coefficient

The EFI, a method that intricately divides twin pairs into two groups based on their age, presents a novel approach that addresses the complexity of environmental influences on DNA methylation. Conversely, the most direct method to explore the connection between DNA methylation and age is to evaluate the linear correlation between methylation levels and chronological age. The EFI was calculated for each probe, and its values are derived using all twin pairs. Therefore, more than a direct comparison between the EFI and chronological age is needed. To address this, we calculated each probe’s correlation coefficient between age and methylation levels and compared these coefficients with the EFI values.

**Figure 1:**
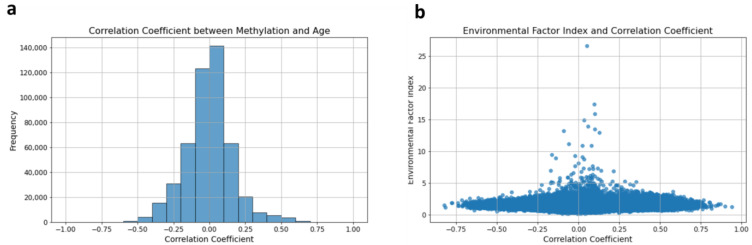
Difference between the EFI and coefficient correlation of DNA methylation values and age: (**a**) distribution of correlation coefficients between methylation values and chronological age for each DNA methylation site; (**b**) scatter plot of EFI and the correlation coefficient. Each point represents a methylation site, plotting its EFI value on the *y*-axis against its corresponding correlation coefficient on the *x*-axis.

**Figure 2:**
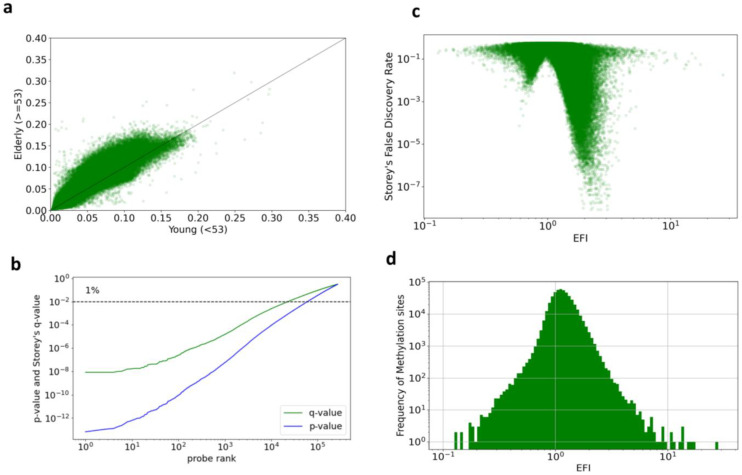
Statistics on EFI. (**a**) Scatter chart of SDs for young twins (<53 years-old) and elderly twins (≥53 years-old). The x- and y-axes correspond to the denominator and numerator of the EFI, respectively. Each dot represents a methylation site. (**b**) *p*-values of Levene’s test and q-values representing the probabilities adjusted for multiple testing correction. (**c**) Scatter chart of EFI and FDR from Levene’s test between young and elderly twins. Each dot represents a methylation site. Both axes are logarithmic. (**d**) Histogram of EFI. Both axes are logarithmic.

Figure 1a presents the distribution of correlation coefficients between DNA methylation levels and chronological age for each DNA methylation site. The distribution of these correlation coefficients is unimodal, indicating a single peak. This characteristic is similar to the unimodal distribution observed in Figure 2d for the EFI values.

Figure 1b plots the EFI values against the correlation coefficients for each methylation site. Each point on the scatter plot represents a methylation site, with the EFI value on the *y*-axis and the corresponding correlation coefficient on the *x*-axis. The lack of a clear relationship between EFI values and correlation coefficients indicates that the EFI captures aspects of the methylation data that are only partially dependent on linear age–methylation relationships.

The differences in DNA methylation levels between twin pairs are primarily explained by the influence of environmental factors. If the influence of environmental factors is neutral, it can generally be assumed that the longer the exposure to the environmental factors (i.e., the older the individual), the greater the differences in DNA methylation levels. This hypothesis is supported by Figure 2a, where 76.5% of the DNA methylation sites show larger differences in the elderly group compared to the younger group.

If the EFI were strongly correlated with age, we would expect a trend where higher correlation coefficients correspond to larger EFI values. However, such a clear trend is not observed in Figure 1b, suggesting that the EFI captures non-linear aspects of age-related methylation changes or other environmental influences beyond simple chronological age.

These results underscore the idea that the EFI and correlation coefficients are fundamentally different metrics. While correlation coefficients measure the strength of the linear relationship between age and methylation at each site, the EFI, with its unique approach of using twin pairs to eliminate the influence of genetic factors, presents a significant distinction from values derived solely from the relationship with age, such as correlation coefficients. This unique aspect of the EFI provides new insights that may be of interest to the scientific community.

### 2.2. Methylation Sites Are Linked to Disorders

The current study was conducted on identical twins to determine the effect of environmental factors on DNA methylation at each site. The results indicated that the degree of methylation increased at 22,568 sites in an age-dependent manner, whereas a decrease was observed at 94 sites. We examined the correlation among significant methylation sites, disease, and CpG.

As detailed in the Introduction, DNA methylation has been reported to be associated with various diseases. Therefore, we examined the link between the significant methylation sites and such disorders. The top 10 sites were identified (Table 1) along with the probe ID on the Infinium HumanMethylation450 BeadChip Kit, the gene symbol of the site, the EFI, and the related disorders. We linked the association between these sites and diseases based on the relationship between the gene symbols of the sites and elite genes from MalaCards [55], which is a database of human diseases (accessed 10 June 2021).

Two methylation sites have been linked to disorders. One of these, known as cg25105066, belongs to the autism susceptibility candidate 2 (*AUTS2*) gene, which is an “Activator of Transcription and Developmental Regulator AUTS2” and an elite gene for “intellectual developmental disorder autosomal dominant 26”. A previous study found that DNA methylation of *AUTS2* is linked to this disorder [56]. Another study discovered that DNA methylation of *AUTS2* in the placenta is associated with neurodevelopment in children [57]. Thus, AUTS2 may be used as a biomarker for autism spectrum disorder risk. The other methylation site, cg14464244, belongs to the *MAGI2* gene (i.e., “Membrane Associated Guanylate Kinase, WW and PDZ Domain Containing 2”). DNA methylation of *MAGI2* has been linked to various cancers [58,59,60], whereas no correlation was established with a nephrotic syndrome.

Table 2 presents the link between disorders and genes strongly influenced by environmental factors. This Table lists the top ten genes with a high number of significant sites. The columns contain the gene symbols, the number of sites, and the associated disorders. We used the elite genes, as defined by MalaCards, to establish the association between genes and disorders (accessed on 10 June 2021).

Table 3 summarizes the findings from Table 1 and Table 2. We identified two sites and seven genes linked to disorders using the elite genes of MalaCards. This Table shows that of the top ten methylation sites selected by EFI, seven were annotated as genes, and among those, two were classified as elite genes. Similarly, seven of the top 10 genes were classified as elite. Based on this, the elite gene ratio for the top ten sites was calculated as 2/7 = 29%, and for the top ten genes, the elite gene ratio was 7/10 = 70%.

As detailed in Section 4.5, we performed Fisher’s exact test, and for the top 10 genes, the null hypothesis was rejected, indicating that the association between the elite genes and disorders was statistically significant.

This analysis suggests that a single site with variable methylation levels resulting from environmental factors is unlikely to be directly related to a disorder. However, when methylation changes are clustered within a single gene, the likelihood of an association with the disorder increases compared with chance, highlighting the potential relevance of our research. Nevertheless, it is essential to acknowledge that methylation changes may also result from the disorder rather than the cause.

### 2.3. Environmental Factors Alter DNA Methylation Levels in Methylation Markers

Genetic markers have been used to detect disorders and can be classified into three categories: (1) biochemical markers are in the blood or other body fluids, indicating the presence of disorders; (2) molecular markers which indicate specific changes or alterations in DNA sequences associated with disease; and (3) methylation markers indicating specific changes in DNA methylation patterns associated with disorders. These markers may be used as diagnostic or prognostic tools as well as potential targets for therapeutic interventions [61,62]. As the methylation levels of these markers alter in response to the environment, they be significant sites for the EFI to accurately capture the environmental factors.

Table 4 lists the number of methylation markers identified for colorectal [19,20,21,63], breast [16,17], cervical [16,18], and lung [22,23,24] cancers, along with the number of genes associated with significant sites. The columns list the total number of biomarkers, the number of genes with significant sites, and the *p*-value obtained from the binomial test. A list of marker genes for each cancer and the number of significant sites is available in the Supporting Information file: Marker.pdf.

A large proportion (85%) of the markers consisted of significant genes, which was higher than the probability of this occurring by chance (31.2%). The results indicated that the EFI can assess the impact of environmental factors on various cancers. The strength of the environmental factors may be evaluated by comparing the variations in the methylation levels between twins.

### 2.4. DNA Methylation on CpG Islands

CpG islands are genome regions containing a high frequency of CpG sites. Approximately 70% of the proximal promoters in humans located near the transcription start site contain CpG islands [64]. Typically, the methylation of CpG islands is associated with transcriptional repression, long-term gene silencing, X-chromosome inactivation, genomic imprinting, and pre-mRNA alternative splicing [65,66]. Recent studies have indicated a regulatory role for DNA methylation [67] and that the methylation of CpG sites in CpG islands alters gene expression [68].

Table 5 lists the information on five different features of CpG methylation, including the total number of methylation sites analyzed using the BeadChip, the number of significant sites, and the ratio of significant sites to the total number of sites. The CpG features were defined as follows: CpG islands include regions >500 bp, >55% GC, and an expected/observed CpG ratio of >0.65. Of note, 40% of the gene promoters contain islands [69], whereas shores are regions located 0–2 kbp from CpG islands and consist of >75% of tissue-specific differentially methylated regions. The methylation in shores is more strongly correlated to gene expression compared with that of the CpG islands [70,71]. Shelves are the 2–4 kbp regions from the islands. North and south indicate upstream and downstream to the CpG island, respectively.

The distribution in Table 5 shows significant methylation sites across different genomic features. This Table shows the number of sites (#sites) and significant sites (#significant sites) for each genomic feature, including North Shelf, North Shore, CpG Islands, South Shore, South Shelf, and Others. The column “% of significant sites in Feature” represents the proportion of significant sites within each feature, calculated by dividing the number of significant sites by the total number of sites in that feature. The column “% of significant sites in total” indicates the proportion of significant sites in the entire dataset, calculated by dividing the number of significant sites in each feature by the total number of probes (481,190). These values allow for a comparison of the distribution of significant sites across various genomic regions.

The proportion of significant sites in the CpG islands was greater than that of the other features, suggesting that CpG islands are highly responsive to environmental factors. Because of their enrichment in regulatory regions, association with tissue-specific gene expression, and susceptibility to DNA methylation changes, CpG islands are essential targets for environmental epigenetic studies and potential biomarkers for disease susceptibility [72]. The findings in Table 5 indicate that the EFI captures the effect of environmental factors on DNA methylation.

## 3. Discussion

In our study, we proposed using the EFI to quantify the impact of environmental factors on DNA methylation from 245 pairs of Japanese monozygotic twins. The EFI calculates the difference in methylation levels between older and younger twin pairs, assuming that the more prolonged lifetime exposure to environmental factors in older twins would result in more pronounced methylation changes. This differential exposure underpins our hypothesis that the more significant the discrepancy in methylation between the age groups, the stronger the environmental influence.

Using Storey’s FDR statistical method, we identified 22,752 out of 481,190 methylation sites on the Infinium HumanMethylation450 BeadChip as environmentally sensitive. The analysis of these sensitive sites, focusing on their association with diseases, cancer methylation markers, and CpG island features, further substantiated the efficacy of the EFI.

Our analysis revealed a significant correlation between environmentally sensitive methylation sites and disease development, underscoring the EFI’s potential to advance our understanding of disease prevention and treatment mechanisms. Furthermore, when evaluating known methylation markers for four types of cancer, we found that 85% of these markers were among the significant sites identified by the EFI, suggesting its robustness in assessing the influence of environmental factors on methylation. The evaluation of CpG island features revealed their heightened sensitivity to environmental factors, highlighting the importance of these regions in environmental epigenetics research and their potential as biomarkers for disease susceptibility.

The challenge of distinguishing between genetic and environmental influences is particularly formidable in epigenetics, where it intersects with the broader objectives of understanding human diseases and developing therapeutic interventions. Historically, twin studies have been instrumental in dissecting the genetic and environmental contributions to phenotypic variance [54,73]. These studies have not only been applied to estimate the impact of these factors on DNA methylation at gene-specific levels, as evidenced by research from Wong [74], but also across the entire genome, as demonstrated in studies by Kuratomi [75], Kaminsky [76], and Rakyan [77]. This body of work has significantly deepened our understanding of the regulatory processes behind epigenetic variation, unraveling the intertwined contributions of epigenetic mechanisms, environmental factors, and genetic variance to complex traits, alongside stochastic elements [53]. The insights gleaned from these studies are invaluable for grasping the intricacies of disease development and evolution [78] and for informing future epigenetic-based strategies to combat complex diseases.

Within this context of rich academic heritage, our study introduces EFI, a novel metric computed using twin subjects. The EFI embodies a significant leap forward, transcending traditional epigenetic inquiries to enhance our grasp of disease mechanisms and potential therapeutic avenues. This methodology allows us to more accurately isolate and quantify the impact of environmental factors on DNA methylation, providing crucial insights that are potentially transformative for identifying the underlying causes of human diseases and crafting new treatment strategies. Our approach thus not only enhances the precision of our epigenetic analysis but offers a powerful tool that transcends traditional research boundaries, promising substantial advancements in medical research and the development of therapeutic interventions.

The present study had some limitations. All the twin subjects were Japanese. Because of the lack of racial and regional diversity with respect to environmental factors, potential data bias may impact the numerator and denominator of the EFI. Predicting the effect of diversity on EFI values remains a challenge. Therefore, collecting data on twins of various races from different regions is essential to determine the effect of environmental factors on the EFI when using diverse populations.

In this study, we divided the twin pairs into two groups based on the median age of 53 years. This decision was made considering several factors, including the distribution of ages among our subjects and the broader context of age distribution, both globally and in Japan.

Firstly, it is crucial to note that average age and median age differ by country. According to the United Nations’ World Population Prospects 2022, the global average age in 2023 is approximately 30.4 years, and the median age is about 31.0 years. For Japan, the average age is 48.4 years, and the median age is 48.6 years. Given that our study’s subjects are from Japan, it was not just pertinent, but a sound and well-grounded decision to align our division with these national statistics. By selecting a median age close to Japan’s average, we ensured a representative and balanced division of our sample.

The choice of the median age is crucial for statistical robustness. It ensures that the number of subjects in each group is approximately equal, which is essential for minimizing bias and enhancing the validity of comparative analyses. We conducted sensitivity analyses using different cutoff points to further validate our choice. We explored divisions at the 25th percentile (35 years) and the 75th percentile (66 years) and found that the correlation coefficients between the EFI values and these age cutoffs were 0.81 and 0.71, respectively. These high correlation values indicate that our findings are relatively sensitive to the exact cutoff point, underscoring the robustness of our methodology.

We acknowledge that differences could influence the Environmental Factor Index (EFI) in terms of cell type composition, a known confounding factor in DNA methylation analyses. Although correction for cell type distribution using leukocyte composition data, such as through the Houseman algorithm [79], is theoretically possible, the dataset used in this study had limitations. Specifically, Osaka Twin Research Group collected the data several years ago, and complete leukocyte composition information was only available for some samples. Consequently, we could not apply cell type correction across the entire dataset. Future studies would benefit from addressing this limitation by incorporating cell type composition data to more accurately assess the specific impact of environmental factors on DNA methylation. Despite this limitation, the EFI offers valuable insights into methylation variability driven by environmental influences while acknowledging the potential confounding effect of cell type distribution.

To explore potential overlaps between the Environmental Factor Index (EFI) and conventional age acceleration measures, such as Horvath’s epigenetic clock algorithms, we analyzed the EFI values associated with the DNA methylation sites used in both the Horvath2013 (353 sites) [35] and Horvath2018 (391 sites) clocks [39]. As shown in Table 6, the average EFI for the Horvath2018 sites was 1.29 with a variance of 0.33, while for the Horvath2013 sites, the average EFI was 1.26 with a variance of 0.34. These averages and variances were higher than those observed in the overall dataset, which included all 481,190 sites (average EFI = 1.18; variance = 0.28). Additionally, the proportion of statistically significant sites within the Horvath clocks was considerably higher than in the overall dataset: 338 out of 391 sites in Horvath2018 and 334 out of 353 sites in Horvath2013 were statistically significant, compared to 22,752 out of 481,190 in the entire dataset.

These results indicate that many of the DNA methylation sites used in the Horvath epigenetic clocks are also significant in the EFI analysis. This overlap is expected, given that the Horvath epigenetic clocks use a linear formula to estimate age, relying on DNA methylation sites whose values are likely to change solely in response to the environmental factor of aging. The fact that the significant sites in the EFI analysis include the sites of the Horvath epigenetic clocks suggests that the EFI is consistent with previous analytical findings. This alignment with established epigenetic age-related sites supports the validity of the EFI in capturing environmentally driven changes in DNA methylation.

One limitation of this study is the need for a more direct analysis linking the CpG sites identified by the Environmental Factor Index (EFI) to diseases or environmental exposure/phenotypes. The dataset used in this study consisted solely of DNA methylation data and did not include detailed participant attributes, such as health conditions or environmental exposure histories. As a result, assessing the direct associations between the EFI and specific diseases or environmental factors was not feasible.

Despite this limitation, the primary objective of this study was to evaluate the variability in DNA methylation caused by environmental factors, and the EFI successfully identified methylation sites exhibiting significant changes. However, to fully understand the implications of these findings, future research should integrate DNA methylation data with detailed participant attributes. Collecting comprehensive data, including participant health conditions and environmental exposure histories, and combining these with disease-related databases will enable a more thorough exploration of the relationships between the EFI and diseases or environmental factors. Such efforts will further elucidate the role of environmental influences on epigenetic regulation and their contribution to disease pathogenesis.

## 4. Materials and Methods

### 4.1. Subjects and Ethics Statement

**Figure 3:**
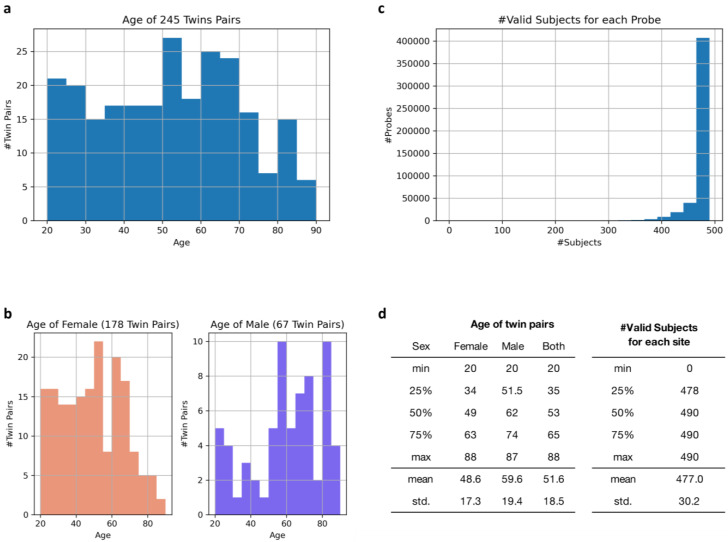
Statistics related to the subjects and data collected for the DNA methylation probes. (**a**) Age distribution of the twin pairs. The *x*-axis represents the age, and the *y*-axis represents the number of pairs. (**b**) Similar histograms showing the age distribution of the twin pairs differentiated by sex. The axes are the same as in (**a**). (**c**) Histogram illustrating how many valid observations were collected for each DNA methylation probe. The *x*-axis shows the number of valid observations, and the *y*-axis represents the number of probes. (**d**) Representative ages of the subjects (the left table) and the number of valid subjects for each probe (the right table). The values in the right table represent basic statistics of DNA methylation probes when sorted in ascending order by the number of valid subjects.

Since 16 January 2011, 302 healthy Japanese identical twin pairs have been recruited to measure DNA methylation. Of these, 245 were monozygotic twin pairs, and the others were dizygotic. We used the monozygotic twin pairs to ensure the genetic uniformity needed for our study. The cohort consisted of 178 pairs of females and 67 pairs of males. The subjects were selected from a registry established by the Center for Twin Research at Osaka University [80,81]. Written informed consent was obtained from all subjects before inclusion in the study. The Ethics Committee of Osaka University approved the study protocol (No. 269). The ages of the subjects are shown in Figure 3a,b. Blood samples were collected from the subjects at 9:00 a.m. after fasting for 12 h. The subjects also underwent a clinical examination and completed a health questionnaire. The examinations were conducted on the same day for each pair of twins. The QIAamp DNA Mini Kit was used to isolate genomic DNA from the peripheral blood mononuclear cells. The zygosity of the twins was confirmed by perfectly matching 15 short tandem repeat loci using a PowerPlex^®^ 16 System (Promega, Madison, WI, USA).

### 4.2. Methylation Sites

DNA methylation levels were analyzed at specific locations in the genome, known as methylation sites. The Infinium HumanMethylation450 BeadChip Kit (Illumina, San Diego, CA, USA) was used to examine 482,421 methylation sites in each sample at single-nucleotide resolution using 0.5 µg of high-quality genomic DNA. The chip consisted of two bead types per locus at each site. The raw data obtained were analyzed using Genome Studio software (Illumina). The fluorescence intensity ratios between the two bead types were calculated as follows: 0 indicated that the site was not methylated and 1 indicated complete methylation. A peak-based correction method [82] was used to normalize the raw data and filter out invalid reads such as null and unreliable probes. Specifically, methylation sites with fewer than 245 valid probes out of 490 subjects were excluded from further analysis, ensuring that only reliable data were retained. After this filtering process, 481,190 methylation sites remained, and their statistics are presented in Figure 3c,d.

### 4.3. Notation

The following notations were used to measure the impact of environmental factors on each methylation site *s*.

m_i,s_: methylation levels of a methylation site *s* in a subject *i*. The site *s* was omitted if it was self-explanatory.

T: a set of twin pairs included in the study. The set is represented as T = {(*i*, *j*)}, where *i* and *j* are the subjects of the twin pair. If (*i*, *j*) is in T, then (*j*, *i*) is not in T.

T*s*: a set of valid twin pairs on methylation site *s*. T*s* is a subset of T, and only included twin pairs in which the methylation levels of site *s* in the subjects *i* and *j* were valid.

D*s*(T*s*): a set of differences in methylation levels of a methylation site *s* between the twins in the twin pair T*s*. This is represented as D*s*(T*s*) = {(m_i,s_ − m_j,s_)|(*i*, *j*) in T*s*}.

SD (D): the standard deviation of a set of differences D.

### 4.4. Environmental Factor Index (EFI)

To determine the strength of the influence of environmental factors on methylation sites, we compared the distribution of methylation intensities between elderly and young twins. We divided the twin pairs (T) into two groups based on the median age of the subject, 53 years. The elderly set (T*_eldery_*) included >=53-year-olds and consisted of 124 twin pairs. The younger set (T*_young_*) included <53-year-old twins and consisted of 121 pairs. Theoretically, the elderly are exposed to environmental factors for longer compared with the young pairs. Thus, the difference between the two groups indicates the degree of influence of environmental factors. We defined the EFI equation to measure this influence on the methylation site.

$$EFI_{s}=\frac{\mathrm{Std}(D_{s}\;(T_{eldery})\;}{\mathrm{Std}(D_{s}\;(T_{young}))}$$

where T*_eldery_* is a set of elderly twin pairs and T*_young_* is a set of younger twin pairs.

The numerator of this Equation represents the distribution of methylation intensity differences in the elderly group, whereas the denominator represents the distribution in the younger group. The period affected by environmental factors is shorter in the denominator and longer in the numerator. Therefore, the EFI is a tool that allows us to deduce the impact of environmental factors on DNA methylation’s variability over time. A higher EFI indicates an amplification of differences due to environmental exposure, while a lower EFI hints at a reduction in these differences.

When EFI*s* = 1, the impact of environmental factors on DNA methylation levels is neutral to age. This means that the variability in DNA methylation differences between twins is the same in the older and younger age groups. When EFI*s* > 1 is greater than 1, this indicates that the variability in DNA methylation differences between twins is more significant in the older age group compared to the younger age group. This suggests that continuous exposure to environmental factors over time amplifies the differences in DNA methylation levels between twin pairs. EFI*s* < 1 indicates that the variability in DNA methylation differences between twins is smaller in the older age group compared to the younger age group. This suggests that continuous exposure to environmental factors over time decreases the differences in DNA methylation levels between twin pairs.

### 4.5. Statistical Analysis of EFI

We aimed to investigate how environmental factors influence DNA methylation in monozygotic twins. To achieve this, we developed the Environmental Factor Index (EFI), which quantifies the variability in DNA methylation levels between younger and older twins, thus isolating the impact of environmental exposure over time. By comparing the differences in methylation intensities between these two groups, the EFI enables us to assess how environmental factors contribute to methylation changes at specific genomic sites.

Figure 2 presents statistics on EFI. Figure 2a shows a scatter chart of the standard deviations (SD) for young and elderly twins. Each dot on the chart represents a DNA methylation site. The line indicates that the values of the two axes are equivalent. Interestingly, 76.5% of the sites are above the line, indicating that the differences in the degree of methylation between the twins increased, specifically reflecting that the standard deviation of methylation in the elderly group is more significant than that in the younger group. The sites below the line indicate a decreasing difference between twins in a time-dependent manner, specifically reflecting that the standard deviation in the younger group is more significant than that in the elderly group.

We conducted a test of the EFI, where the denominator is the SD of young twin pairs and the numerator is the SD of elderly twin pairs. Therefore, to test the EFI, we utilized Levene’s test, which assesses the equality of variances between the two groups, to examine whether there is a significant difference between the standard deviations of these two populations. Specifically, we formulated a null hypothesis that the variances in the denominator and numerator are equal. We performed these calculations using Python 3, specifically employing the SciPy library for Levene’s test. Given that 481,190 DNA methylation sites are subject to testing, it is necessary to address the issue of multiple comparisons. Therefore, we applied Storey’s FDR approach [83]. To determine the significance of the methylation sites, we used a significance level of 1%.

Figure 2b shows the results of Levene’s test for each methylation site using the multiple testing correction. The line represents the probability of the test after the correction of Storey’s FDR (*q*-value). The dotted horizontal line indicates the significance level of 1%. The number of sites in which the null hypothesis was rejected was 22,752 and the ratio to the total number of observed methylation sites (481,190 sites) was 4.7%. For convenience, we refer to the methylation sites in which the null hypotheses were rejected by Storey’s FDR as significant sites.

To validate the EFI statistically, we performed Levene’s test to compare the variability in DNA methylation levels between younger and older twins. This analysis determines whether the observed differences in methylation variability are statistically significant, thus confirming the influence of environmental factors on DNA methylation over time.

Figure 2c shows a scatter chart of the EFI and Storey’s FDR results, analyzed using a Levene’s test, for young and elderly twins. Each dot represents a methylation site, with logarithmic scales used for the x- and y-axes. A depression is evident in the chart at EFI = 1. Because the denominator and numerator of the EFI are identical at this value, the *p*-value for Levene’s test is necessarily close to 1. In this scatter chart, methylation sites with a small FDR are more prevalent on the right side of this Figure, where EFI > 1. Of the 22,752 methylation sites identified as significant, 22,568 had an EFI > 1, whereas only 94 sites had an EFI < 1. For our interpretation, we designated the significant sites with EFI values > 1 as “elderly-significant sites” and those with <1 as “young-significant sites”.

The histogram in Figure 2d shows the EFI distribution with logarithmic x- and y-axes. Of the 481,190 methylation sites, 112,868 (23%) had an EFI < 1, and 368,322 (77%) sites had an EFI>1. The median EFI value was 1.137, indicating that the difference in the degree of DNA methylation between twins may increase over time.

### 4.6. Statistical Analysis of Disease Association

We identified the methylation sites and associated genes selected by the EFI and investigated their relationship with disorders using the MalaCards database. Following this, we performed a coincidence test to confirm that the associations discovered by the EFI were not due to random chance. The null hypothesis stated that the elite genes appeared by chance.

MalaCards is a comprehensive database that compiles information on human diseases, including genetic associations, pathways, and relevant research. Some of the genes listed in MalaCards are classified as “elite genes”. These elite genes, which we found to be of the utmost importance, are genes that have strong evidence linking them to specific diseases based on high-confidence sources such as curated databases, clinical studies, or genome-wide association studies (GWAS). The “elite” designation indicates that these genes are strongly associated with certain disorders, making them important candidates for further investigation in disease-related studies.

The MalaCards database does not publish the number or ratio of elite genes, which is necessary information for the coincidence test. Therefore, we independently gathered the relevant data by randomly sampling 100 sites from an array of 481,190 sites on BeadChip. To assess the statistical significance of this association, we applied a two-tailed Fisher’s exact test with a significance level of 1%.

## 5. Conclusions

Our study has pioneered use of the EFI, marking a significant stride in the epigenetic domain by quantifying the environmental impacts on DNA methylation with unparalleled precision. This novel metric utilizes an extensive dataset derived from 245 monozygotic twin pairs. It examines 481,190 methylation sites, establishing a robust framework that enhances our understanding of the intricate relationship between the environment and the epigenome.

The identification of 22,752 DNA methylation sites significantly influenced by environmental factors using the Environmental Factor Index (EFI) highlights the tool’s sensitivity and capacity to elucidate the complex dynamics of epigenetic modifications. This accomplishment is particularly significant given the long-standing challenge of detecting these subtle influences within the complex epigenome. Moreover, the analysis conducted with the EFI reveals that although individual environmentally sensitive methylation sites might only slightly impact phenotypic traits, their aggregated effect within genes significantly correlates with phenotypic expression. This insight enriches our understanding of the cumulative influence of the environment on gene regulation and expression.

Moreover, by applying the EFI to the Malacards database, our study illuminated the potential connections between environmentally influenced methylation sites and various disorders, notably cancer, suggesting the EFI’s utility in identifying epigenetic markers of disease. This application validates the EFI’s relevance and opens new research avenues for exploring the epigenetic underpinnings of various diseases and conditions.

The introduction of the EFI represents a transformative development in epigenetic research, offering a new lens through which we can explore the complex interplay between the genome, the epigenome, and environmental factors. As we move forward, the EFI stands to significantly enhance our understanding of epigenetic regulation, shed light on the mechanisms underlying disease, and inform the development of novel diagnostic and therapeutic approaches. Our findings affirm the potential of the EFI as a cornerstone in future epigenetic investigations, heralding a new era of precision in quantifying environmental influences on the epigenome.

## Figures and Tables

**Table 1:** Top ten methylation sites in the EFI.

Probe ID	Gene Symbol	EFI	q-Value	Disorder
cg11539424	*CLGN*	5.22	0.057%	
cg25105066	*AUTS2*	5.14	0.0055%	Intellectual Developmental Disorder, Autosomal Dominant 26
cg14464244	*MAGI2*	4.76	0.047%	Nephrotic Syndrome, Type 15 Genetic Steroid-Resistant Nephrotic Syndrome
cg06445586		4.71	0.31%	
cg02878907	*ZNF709*	4.65	0.034%	
cg04883656	*OGFRL1*	4.34	0.023%	
cg21155461	*ZNF544*	4.04	0.68%	
cg21364278		4.01	0.37%	
cg17289202	*ZNF532*	3.95	0.18%	
cg15368722		3.86	0.084%	

**Table 2:** Top ten genes in the EFI.

Gene Symbol	#Significant Site	Disorder
*PTPRN2*	39	
*TNXB*	34	Ehlers–Danlos Syndrome, Classic-Like Vesicoureteral Reflux 8
*PRDM16*	31	Left Ventricular Noncompaction 8
*BRUNOL4*	28	
*COL11A2*	25	Otospondylomegaepiphyseal Dysplasia, Autosomal Dominant/Recessive
*NKX6-2*	24	Spastic Ataxia 8, Autosomal Recessive, with Hypomyelinating Leukodystrophy
*PCDHGA4*	21	
*THRB*	20	Thyroid Hormone Resistance, Generalized, Autosomal Dominant Thyroid Hormone Resistance, Selective Pituitary
*MAGI2*	20	Nephrotic Syndrome, Type 15 Genetic Steroid-Resistant Nephrotic Syndrome
*TP73*	20	Small Cell Cancer of the Lung

**Table 3:** Statistical tests for predominantly high levels of disease-related genes.

	Top 10 Sites	Top 10 Genes	Random
#Sites	10	-	100
#Genes	7	10	77
#Elite genes	2	7	19
Elite gene ratio	29%	70%	25%
*p*-value	1.000	0.0067	-
Odds ratio	0.86	7.12	-

**Table 4:** Statistical tests for predominantly high levels of disease-related genes.

	#Markers	#Significant Sites	Ratio	*p*-Value
Colorectal	51	39	76%	3.80 × 10^−11^
Breast	11	11	100%	2.71 × 10^−6^
Cervical	7	7	100%	2.87 × 10^−4^
Lung	16	15	94%	2.91 × 10^−7^
Total	85	72	85%	2.11 × 10^−24^

**Table 5:** Significant sites for each CpG feature.

			% of Significant Sites	
Feature	#Sites	#Significant Sites	in Feature	in Total
North Shelf	24,716	801	3.2%	0.17%
North Shore	62,647	2422	3.9%	0.50%
CpG Island	149,935	10,898	7.3%	2.3%
South Shore	49,055	1850	3.8%	0.38%
South Shelf	22,182	712	3.2%	0.15%
Other	172,655	6069	3.5%	1.3%
Total	481,190	22,752	4.7%	4.7%

**Table 6:** EFI of the Horvath 2018 clock sites and the Horvath 2013 clock sites.

	Horvath 2018	Horvath 2013	All Sites
# sites	391	353	481,190
# significant sites	338	334	22,752
Mean of EFI	1.29	1.26	1.18
Std. of EFI	0.33	0.34	0.28

## Data Availability

The methylation levels of each site for each subject that support the results of this study are not publicly available. The small number of twin participants, coupled with the unique identifying nature of twin status, facilitates easier identification of individuals. Therefore, due to the Center for Twin Research’s policies to protect the individual’s privacy, the data have not been made publicly accessible. However, upon reasonable request, data may be obtained with the permission of the Center for Twin Research, Osaka University Graduate School of Medicine (research@twin.med.osaka-u.ac.jp).

## Supplementary Materials

The following supporting information can be downloaded at: https://www.mdpi.com/article/10.3390/epigenomes8040044/s1, Marker.pdf, EFI.xlsx, EFGene.csv. Marker.pdf: The file includes DNA methylation marker genes for four cancers and the number of EFI significant sites. EFI.xlsx: The file includes the EFI and Storey’s FDR for each of the 481,190 methylation sites. Each methylation site has a probe ID of the HumanMethylation450 BeadChip (GPL 18809 @ Gene Expression Omnibus) and a gene symbol. The csv file has four columns, as follows: 1. Probe ID; 2. Gene symbol; 3. EFI; 4. FDR. EFIGene.csv: The file includes environmentally sensitive gene symbols. The file has three columns, as follows: 1. Gene symbol; 2. Number of elderly significant probes; 3. Number of young significant probes.
