# Supplementary material for: Environmental Factor Index (EFI): A Novel Approach to Measure the Strength of Environmental Influence on DNA Methylation in Identical Twins
Source: Epigenomes. 2024 Nov 21;8(4):44. doi: 10.3390/epigenomes8040044 (PMC11587003; doi:10.3390/epigenomes8040044)
Supplement: Supplementary file 1 [file epigenomes-08-00044-s001.zip › Marker.pdf]

**a**

**Colorectal**

| Marker | #sites |
|---|---|
| ALX4 | 15 |
| APC | 1 |
| BRAF | 1 |
| CACNA1G | 0 |
| CAV1 | 5 |
| CD133 | 5 |
| CDH1 | 1 |
| CDH13 | 4 |
| CDH3 | 7 |
| CDKN2A | 3 |
| CRBP1 | 0 |
| DAPK | 1 |
| DFNA5 | 0 |
| DKK1 | 6 |
| ESR1 | 5 |
| GATA4 | 8 |
| GATA4 | 8 |
| GATA5 | 14 |
| GSTP1 | 1 |
| HACE1 | 1 |
| HIC1 | 0 |
| HLTF | 0 |
| HTR6 | 2 |
| ID4 | 2 |
| IGF2 | 0 |
| IRF8 | 0 |

**Colorectal**

| Marker | #sites |
|---|---|
| ITGA4 | 0 |
| KRAS | 0 |
| MAP1B | 4 |
| MGMT | 1 |
| MINT1 | 0 |
| MINT31 | 5 |
| MLH1 | 2 |
| MYOD | 1 |
| NDRG4 | 5 |
| NGFR | 2 |
| NTN1 | 1 |
| OSMR | 3 |
| RASSF1A | 2 |
| RIZ1 | 4 |
| RUNX3 | 2 |
| SEPT9 | 2 |
| SFRP1 | 8 |
| SFRP2 | 8 |
| SFRP4 | 0 |
| SLC5A8 | 2 |
| THBS1 | 10 |
| TIMP3 | 1 |
| TMEFF2 | 1 |
| UCHL1 | 4 |
| VIM | 0 |

**b**

**Breast**

| Marker | #sites |
|---|---|
| ADCY8 | 3 |
| APC | 1 |
| C1QL2 | 6 |
| DPYS | 6 |
| GRIK1 | 3 |
| IRF4 | 1 |
| KCNQ2 | 2 |
| RASSF1A | 2 |
| SKOR2 | |
| SLC9A3 | 9 |
| SYNDIG1 | 6 |
| TERT | 2 |

**c**

**Cervical**

| Marker | #sites |
|---|---|
| CDH1 | 4 |
| CDH13 | 7 |
| GABRA2 | 5 |
| MYOD1 | 5 |
| RAB3C | 3 |
| SLC5A8 | 2 |
| ZNF257 | 1 |

**d**

**Lung**

| Marker | #sites |
|---|---|
| BCAT1 | 0 |
| CDO1 | 3 |
| CMTM2 | 7 |
| CMTM2 | 7 |
| CPLX2 | 11 |
| DPP6 | 15 |
| GRIK2 | 5 |
| HOXD3 | 7 |
| LVRN | 6 |
| LYPD5 | 5 |
| MDFI | 3 |
| PAX3 | 14 |
| RPL39L | 1 |
| TBX4 | 1 |
| TRIM58 | 7 |
| ZNF177 | 4 |
